# The Effect of Huntington’s Disease on the Basal Nuclei: A Review

**DOI:** 10.7759/cureus.24473

**Published:** 2022-04-25

**Authors:** Olivia C Matz, Muhammad Spocter

**Affiliations:** 1 Anatomy, Des Moines University, Des Moines, USA

**Keywords:** basal nuclei, degeneration, anticipation, gaba, intrinsic loops, autosomal dominant trinucleotide, striatum, chorea, basal ganglia, huntington's disease

## Abstract

Huntington’s disease is an autosomal dominant trinucleotide repeat disorder that causes the progressive degeneration of the basal nuclei. This degeneration leads to clinical symptoms affecting voluntary movement, cognitive impairment, and psychiatric disorders. The patient affected by this disease demonstrates anticipation, meaning that even though there is normal embryological development, the signs and symptoms appear at an earlier age as the gene is continually passed throughout subsequent generations. The degeneration occurs due to the accumulation of the protein *Huntingtin *that destroys the medium spiny neurons located in the caudate and putamen, collectively termed the striatum. Four pathways converge onto the striatum known as the “input” center. These consist of the motor loop, oculomotor loop, association loop, and limbic loop. In each of these loops, the striatum maintains an inhibitory gamma-aminobutyric acid (GABA)-ergic function. The imbalance of the inhibitory versus excitatory input directly relates to the symptoms seen in Huntington’s disease such as the inability to control voluntary movements termed chorea, the inability to control voluntary saccadic ocular movements, the cognitive inability to plan and determine the direction of movement, and the inability to control the emotional and motivational aspects of the movement. There is currently no cure for Huntington’s disease but there is a symptomatic treatment for the chorea and psychiatric conditions. Further research is being done to determine the pathophysiology behind the *Hungtintin* protein to allow for a targeted treatment regimen while also looking into reliable biomarkers for the progression of Huntington’s disease.

## Introduction and background

Huntington’s disease is an autosomal dominant trinucleotide repeat disorder that was first described by George Huntington in 1872 [1]. The repeated segments of cytosine-adenine-guanine (CAG) on chromosome four lead to progressive degeneration of the basal nuclei, specifically of the medium-sized densely spiny neurons found within the caudate and putamen which are collectively called the striatum. As the basal nuclei degenerate, the ability to function as a relay center between external stimuli to the cerebral cortex is lost. This disorder affects 2.7 per 100,000 individuals worldwide with Europe having the highest rate of 10 per 100,000 individuals [1] and has a broad impact on their functional abilities in voluntary movement, cognition, and psychiatric well-being. Specific symptoms include chorea, subcortical dementia, depression, and obsessive-compulsive disorder. Once the symptoms appear, the individual has approximately 10 to 20 years of remaining longevity. In the upcoming pages, there will be a discussion of the anatomy, embryology, histology, and neuroanatomical pathways that occur within the normal functioning basal nuclei and then the dysfunctional basal nuclei of Huntington’s disease.

## Review

The basal nuclei

The basal nuclei are clusters of cell bodies found deep within the telencephalon of the cerebral hemispheres that carry out a multitude of functions. These functions range from motor control, cognition, saccadic eye movements, and facial expressions. The structures within the basal nuclei include the caudate and putamen (striatum) and the globus pallidus interna and externa. The substantia nigra is found within the mesencephalon and the subthalamic nucleus within the diencephalon [2]. The nuclei can be functionally categorized as input, intermediate, and output nuclei. The input nuclei are the striatum which receives signals from the cerebral cortex. The intermediate nuclei are the subthalamic nucleus, globus pallidus externa, and substantia nigra pars compacta which relay signals between the input and output nuclei. The output nuclei are the substantia nigra pars reticulata and the globus pallidus interna which send information to the thalamus which in turn transmits a final signal back to the cortex to complete the circuit [3].

Embryology

The basal nuclei demonstrate a complex embryologic pattern of development which correlates to the complex functions it carries out in an adult individual. In the developing embryo, the basal nuclei originate from within the ectodermal tissue of the neural plate. By day 18, the neural groove appears in the midline of the neural plate. As the groove invaginates, the neural folds at the edges of the neural plate elevate dorsally and fuse in the midline to form the neural tube. Two days later, the primordia of the three primary brain vesicles (the prosencephalon, mesencephalon, and rhombencephalon) can be observed at the cranial end of the neural tube whereas the remaining caudal portion of the neural tube forms the rest of the spinal cord [4]. Further divisions occur as the prosencephalon (forebrain) divides into the telencephalon and diencephalon whereas the rhombencephalon (hindbrain) divides into the metencephalon and myelencephalon [4]. The mesencephalon remains in the middle of the midbrain without any further divisions. The telencephalon further divides around day 40 into the pallidum and the sub-pallidum. The pallidum turns into the cerebral cortex whereas the sub-pallidum forms two transient swellings called the medial and lateral ganglionic eminences which are separated by the sulcus limitans. The lateral ganglionic eminence gives rise to the striatum and the globus pallidus externa whereas the medial ganglionic eminence gives rise to the globus pallidus interna [5]. The substantia nigra develops from the mesencephalon (midbrain) and the subthalamic nucleus from the diencephalon (forebrain) [2]. The structure of the basal nuclei is formed before birth, but functionality is not achieved until myelination occurs during the first year of life [4].

Anatomy and histology

The Striatum

Each portion of the basal nuclei plays an important role in the circuitry that allows an individual to move, react, and express themselves. The largest input component of the basal nuclei is the striatum (caudate and putamen nuclei) which has a volume of approximately 10 cubic centimeters [2]. The putamen, as well as the globus pallidus interna and globus pallidus externa, are included in a group called the lenticular nuclei. The globus pallidus interna is considered an output nucleus and the globus pallidus externa is considered an intermediate nucleus. The striatum receives a blood supply from acutely angled branches of the middle cerebral artery called the lenticulostriate arteries. The acute angles of these vessels cause the striatum to be extremely vulnerable to hemorrhage or emboli occlusion in patients with uncontrolled hypertension. The nucleus accumbens and the olfactory tubercle have morphological and anatomical similarities to the striatum. Due to these similarities, they are considered extensions of the striatum and are implicated in reward learning and addiction [3].

The caudate nucleus is a C-shaped structure that contains a head, body, and tail located on the lateral wall of the lateral ventricles. The lenticular nuclei, which were defined previously, are a lens-shaped group of cell bodies surrounded by the caudate and thalamus medially with the external capsule laterally [2]. All of these nuclei are categorized as lenticular nuclei because they have similar histological, neurochemical, and connectional characteristics.

The predominant neurons found within the striatum are termed medium-sized densely spiny neurons which receive input from the cortex and relay messages to the globus pallidus and thalamus [6]. These medium-sized densely spiny neurons receive modulatory dopaminergic and excitatory glutamatergic input allowing for the release of the inhibitory neurotransmitter gamma-aminobutyric acid (GABA) and specific peptides based on the location within the striatum. The activation of specific receptors of the medium-sized densely spiny neurons leads to antagonistic actions seen within the two pathways of the striatum called the direct and indirect pathways. Striatal medium-sized densely spiny neurons that project via the direct pathway contain type 1 dopaminergic receptors that lead to the activation of adenylate cyclase and further release of Substance P. The striatal neurons that project via the indirect pathway express type 2 dopamine receptors that inhibit adenylate cyclase and cause the release of enkephalin [3]. The details of these pathways will be discussed further under neuroanatomy. 

The Substantia Nigra

 The striatum receives excitatory glutaminergic input from the cerebral cortex and modulatory dopaminergic input from the substantia nigra. The substantia nigra, which is located in the midbrain, consists of two vastly different nuclei: the pars compacta which projects dopaminergic nigrostriatal fibers to the striatum, and the pars reticulata which projects GABA-ergic nigrothalamic fibers to the thalamus. The pars compacta influences the striatum and is classified as an intermediate nucleus. The pars reticulata, which resembles the globus pallidus internus in function, inhibits the ventral anterior and ventral lateral nuclei of the thalamus and is classified as the output nucleus [2]. The neurons within the substantia nigra contain tyrosine hydroxylase which is the first enzyme for the biosynthesis of dopamine. A byproduct of this pathway is neuromelanin which leads to the dark color and the derivation of the name substantia nigra [3]. As mentioned previously, dopamine is released from the substantia nigra and depending on which dopaminergic receptor it encounters in the striatum, this will determine the effect on the basal nuclei circuits. Activated D1 receptors release Substance P for the direct pathway and activated D2 receptors release Enkephalin for the indirect pathway.

The Subthalamic Nucleus

The subthalamic nucleus, which is classified as an intermediate nucleus, is located ventral to the thalamus and rostral to the substantia nigra. The subthalamic nucleus is considered part of the indirect pathway as the nucleus excites the globus pallidus internus via long, sparsely spiny glutaminergic connections that will further inhibit the thalamus. This pathway is considered the traditional circuitry model of the basal nuclei. Further research has shown that the subthalamic nucleus has another role in a revised pathway termed the “hyperdirect pathway”. The hyperdirect pathway allows the motor, premotor, and frontal cortex to provide direct input to the subthalamic nucleus and completely bypass the basal nuclei [3]. The hyperdirect pathway demonstrates that the subthalamic nucleus can mediate a quick cortical excitation of the indirect inhibitory output basal nuclei. The quick excitation of the subthalamic nucleus leads to an immediate halt of an action.

Neuroanatomy

**Figure 1:**
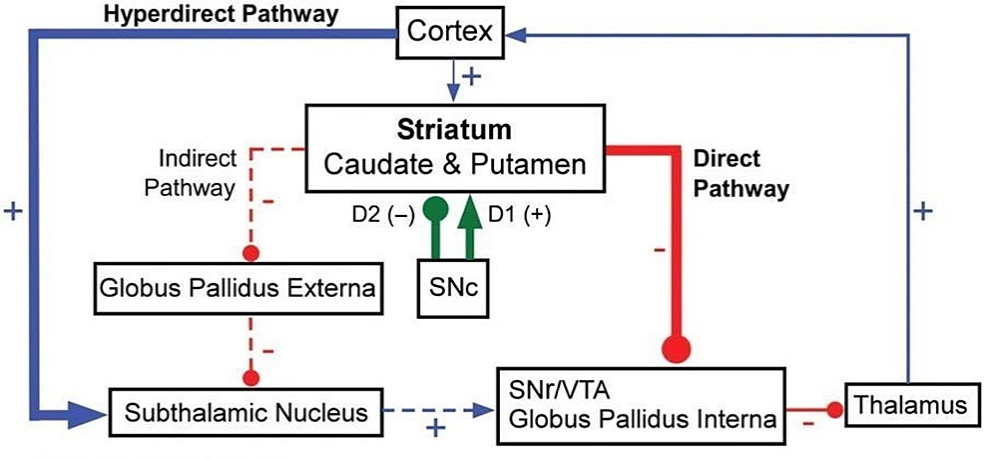
Direct and indirect pathways of the basal nuclei SNc: Substantia nigra compacta, SNr: Substantia nigra reticulata, VTA: Ventral tegmental area Excitatory indicated by the blue line, glutamine; Inhibitory indicated by the red line, gamma-aminobutyric acid (GABA)/Substance P/enkephalin; Modulatory indicated by the green line, dopamine. Image modified from Figure 5 in [7].

This section provides an in-depth discussion of the neuroanatomical function of the basal nuclei with a special focus on the striatum. The striatum plays a pivotal role within the intrinsic circuitry of the brain relating to the direct and indirect thalamic pathways seen in Figure 1 while also being a relay center for the four major pathways that take external stimuli and formulate a reaction. 

Intrinsic Circuitry

In the direct intrinsic pathway, the cerebral cortex receives information from the body. For an individual to respond to an external stimulus, the cerebral cortex sends a transient excitatory glutamatergic signal to the striatum. Using medium-sized densely spiny neurons that contain Substance P and GABA, the striatum causes an inhibition of the globus pallidus interna and the substantia nigra. These two areas tonically release GABA, which inhibits the ventral anterior and ventral lateral thalamus. When the striatum releases a transient inhibitory signal to the globus pallidus interna and substantia nigra, it will disinhibit or momentarily free the ventral anterior and ventral lateral thalamus. The thalamus is now able to excite the motor cortex which ultimately promotes movement using corticospinal projections [2]. Positive feedback is also seen from the thalamus as it further stimulates the Substance P and GABA neurons in the striatum. This pathway is termed "direct" because the striatum is directly inhibiting the globus pallidus interna and substantia nigra to allow movement to occur [7]. 

In the antagonistic indirect pathway, the cerebral cortex starts the circuit by sending a transient excitatory glutamatergic signal to the striatum. Instead of going to Substance P neurons, the signals travel to GABA and enkephalin containing neurons that are located within the striatum. The inhibitory signal travels to the globus pallidus externa, which under normal circumstances tonically releases inhibitory GABA to the subthalamic nucleus. The incoming signal from the striatum and globus pallidus externa inhibit each other so the subthalamic nucleus can transiently release an excitatory glutamatergic signal to the globus pallidus interna and substantia nigra. Unlike the direct pathway, the indirect pathway increases the ability to inhibit the ventral anterior and ventral lateral thalamus and ultimately decreases motor function. To summarize both pathways (as seen above in Figure 1), pauses of neuronal firing through the direct pathway are associated with an action whereas discharges of neuronal firing through the indirect pathway are associated with cessation of movement [7]. 

Motor Loops

The intrinsic circuits mentioned previously are not the only pathways that rely on the basal nuclei. The basal nuclei are considered a relay station for four major loops that are functionally subdivided into the motor, oculomotor, association, and limbic loops [8]. Each of these circuits integrates information in a similar loop pattern from the cerebral cortex to the basal nuclei, the thalamus, and finally back to the unique areas of the frontal cortex where the motor activity is initiated. Each of the following paragraphs will describe the details of these major loops.

The motor loop allows signals to flow from the supplementary motor cortex, somatosensory cortex, primary motor cortex, and premotor cortex to the caudate and putamen in the striatum. These signals travel to the globus pallidus and substantia nigra, then the thalamus, and finally back to the supplementary motor cortex in the frontal lobe. In the frontal lobe, these signals are sent to the upper motor neurons of the cortico-nuclear and corticospinal tracts to influence movement via lower motor neurons. This pathway allows the planning of actions, so an individual can execute a sequence of learned motor activities [8].

The oculomotor loop integrates information from the frontal eye field, prefrontal cortex, and posterior parietal cortex. The signals travel through the caudate in the striatum, then globus pallidus, thalamus, and eventually back to the frontal eye field to create a closed loop. This loop is used when there is a sudden appearance of an object in one’s visual field. The sudden appearance triggers a rapid simultaneous rotation of both eyes known as a saccadic ocular movement [9].

The association loop allows the prefrontal cortex, premotor cortex, and posterior parietal cortex to converge onto the caudate nucleus in the striatum. The pathway continues like the others through the globus pallidus, then the thalamus, and eventually back to the prefrontal cortex. This loop is known as the cognitive or executive loop because it carries the main function of planning motor activities and determining the direction of movement [8]. 

The final loop is the limbic loop, in which the anterior cingulate gyrus, medial and lateral temporal lobe, hippocampus, amygdala, and the entorhinal area all send sensory input to the caudate nucleus which from there travels to the globus pallidus, then thalamus and back to the anterior cingulate gyrus [8]. Each portion sends signals into the basal nuclei to allow emotional and motivational aspects of movement to occur. These aspects can include facial expressions and reward-seeking behaviors.

Patient presentation

The basal nuclei are a complex cluster of cell bodies that when damaged, create a significant and disruptive pattern in an individual’s daily life. Huntington’s disease, one of the first autosomal dominant trinucleotide repeat disorders discovered, affects 2.7 per 100,000 individuals worldwide with Europe having the highest rate of 10 per 100,000 individuals [1]. The trinucleotide repeats of CAG on chromosome four cause an accumulation of the toxic protein, *Huntingtin*, that initiates the progressive degradation of the striatum. The clinical signs typically appear around 20 to 65 years of age if the individual has complete penetrance. Complete penetrance is classified as an individual having greater than 36 CAG repeats on chromosome four. The number of repeats is documented using linkage analysis and can be done at any time of an individual’s life starting prenatally at 10 to 12 weeks gestation [1]. If an individual has less than 36 repeats, they are considered a carrier of the mutated chromosome with incomplete penetrance of the diagnostic symptoms.

As the disease is passed down to each generation, the genetic phenomenon called anticipation is observed as the disease occurs at an earlier age with greater severity due to the larger accumulation of genomic repeats [1]. This explains why parents who do not show symptoms of Huntington’s disease could have a child with the clinical symptoms. To make the diagnosis of Huntington’s disease, a thorough patient and family history must be done. While motor changes and strong family history are enough for a diagnosis, the classic clinical presentation of motor, cognitive, and psychiatric disturbances are all frequently seen at presentation [10]. These classic symptoms will be discussed after looking at the pathology and anatomical changes that occur during the pathogenesis of Huntington’s disease.

Pathology of mutated *Huntingtin*

Chromosome four encodes for the protein *Huntingtin* which is normally found in all neurons and glial cells within the brain and is hypothesized to be involved with internal cellular signalling, preventing apoptosis, intracellular/intercellular transport, and producing brain-derived neurotrophic factor (BDNF). While it is known that the accumulation of mutated *Huntingtin* leads to striatal atrophy, the exact disease-causing mechanism of the mutated protein is currently unknown. The common hypothesized pathway is that the CAG repeats lead to an expanded stretch of glutamine at the amino-terminal end of the protein. This allows the mutated *Huntingtin* to accumulate as abnormal inclusion bodies within the axon, nucleus, and cytoplasm of neurons and glial cells. Autophagy normally occurs when a protein is damaged or formed incorrectly by degradation mechanisms such as ubiquitin-mediated degradation or lysosomal-autophagy degradation. With the mutated *Huntingtin*, the protein is resistant to degradation which causes an accumulation within the different cellular compartments and a compensatory upregulation of autophagy over time. Many neurons and glial cells can compensate for the accumulation by increasing the chaperone protein load and protein degradation machinery but in the basal nuclei, the striatal medium-sized densely spiny neurons lack this compensatory ability. The inability to adapt to the extra stress of mutated *Huntingtin* accumulation is hypothesized to lead to the progressive neurotoxicity and atrophy of the striatum [10]. 

Anatomical changes

Motor Changes

**Figure 2:**
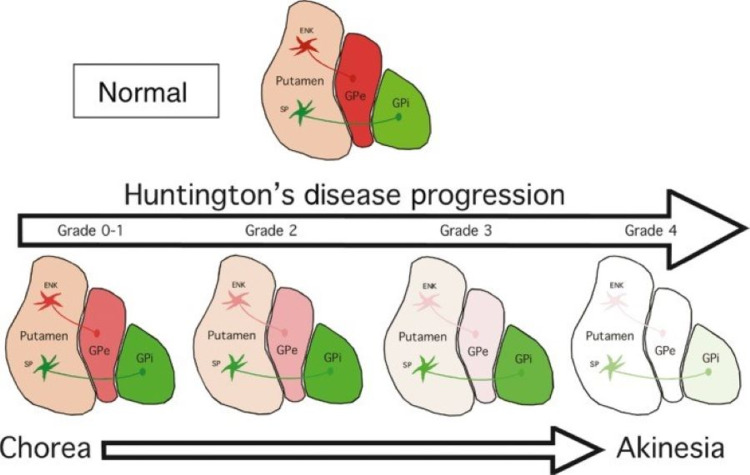
Degenerative process of the striatum in Huntington’s disease ENK: Enkephalin containing neurons of the indirect pathway, SP: Substance P containing neurons of the direct pathway, GPe: Globus pallidus externa, GPi: Globus pallidus interna Image from Figure 7 in [11]. Reproduced after seeking written permission from John Wiley and Sons.

As mentioned in the above section titled 'Intrinsic Circuitry', the basal nuclei function via the direct and indirect pathways. The direct pathway uses GABA and Substance P containing neurons whereas, the indirect pathway uses GABA and enkephalin containing neurons. The accumulation of mutated *Huntingtin* has a detrimental effect on the medium-sized densely spiny neurons of both pathways with the indirect pathway having the greatest vulnerability. The progression of the disease, which is observed and diagnosed clinically, can be seen in Figure 2 with a visual demonstration of the grades and symptoms seen with Huntington’s disease.

**Figure 3:**
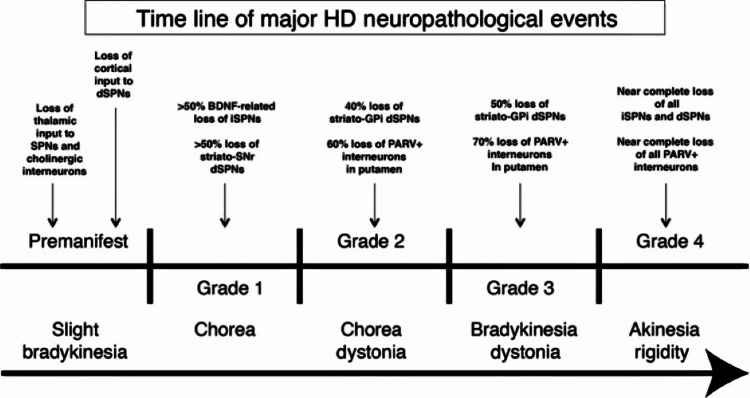
Timeline of neuropathological events of Huntington's disease SPN: Striatal projection neurons, dSPN: Direct pathway striatal projection neurons, BDNF: Brain-derived neurotrophic factor, iSPN: Indirect pathway striatal projection neurons, SNr: Substantia nigra reticulata, GPi: Globus pallidus interna, PARV: Parvalbumin Image from Figure 16 in [11]. Reproduced after seeking written permission from John Wiley and Sons.

As Huntington's disease progresses, the patient goes through different grades of the disease as seen in Figure 3. Grade Zero is considered the premanifest stage where the individual has the mutation, but they are asymptomatic. The symptoms usually occur around 20 to 65 years of age due to the accumulation of mutated *Huntingtin* reaching toxic levels. The more CAG repeats an individual has on chromosome four, the earlier the accumulation reaches a toxic level. In Grade One, the earliest motor abnormalities seen are saccadic eye movements. In this stage, 50% of the indirect medium-sized densely spiny neurons have been lost. In Grade Two, the indirect pathway degenerates further which gives rise to the characteristic “chorea” seen in Huntington’s disease. These are involuntary and unwanted movements that can appear almost dancelike, which become more disruptive as the striatum continues to atrophy. In an individual with normal basal nuclei function, the indirect pathway would inhibit these unwanted movements but due to the greater vulnerability of the indirect pathway, the direct pathway begins to hyperfunction as an unbalanced circuit. The hyperactive direct circuit causes an increased inhibitory stimulation of the globus pallidus interna which frees the ventral anterior and ventral lateral thalamus to cause increased excitation of the motor cortex. At first, the excess movement occurs in the distal extremities of the fingers, toes and small facial muscles [1]. Through observation of the individual, these movements may be imperceptible or be interpreted as fidgeting or nervousness.

As the disease progresses to Grade Three, the patient’s walking becomes unstable and the chorea eventually spreads to all muscles. In Grade Three, there is a greater than 50% loss of the medium-sized densely spiny neurons associated with the direct pathway. The implication of losing neurons within the direct pathway is that there is a severe hesitation in initiating movement which is also a major symptom of Parkinson’s disease. As Huntington’s disease progresses to Grade Four, there is a near-complete loss of all the direct and indirect medium-sized densely spiny neurons in the striatum which causes a complete loss of voluntary movement control, rigidity, dysarthria, and dysphagia [11].

Psychiatric Changes

The psychiatric symptoms of Huntington’s disease present early in the disease progression and are often seen before the motor symptoms. The most common psychiatric condition is depression, but apathy, anxiety, and obsessive-compulsive disorder have also been described. In an individual with normal basal nuclei function, the basal nuclei receive input from the anterior cingulate gyrus, medial and lateral temporal lobes, hippocampus, amygdala, and the entorhinal area to maintain the limbic loop of motivationally and emotionally charged movement [8]. When the striatum begins to atrophy, these extrinsic stimuli are not processed at their target which leads to the development of psychiatric disorders. The new onset of obsessive-compulsive disorder can be very disturbing for the patient and often leads to the development of irritability and aggression. This can range from unnecessary disputes to extreme physical aggression [1]. While obsessive-compulsive disorder is the most disturbing for patients, depression is most commonly described and is seen in 40% to 70% of patients [12]. Due to the large percentage of patients with depression, suicide is the second most common cause of death behind aspiration pneumonia [12]. 

Cognitive Changes

In Huntington’s disease, the cognitive changes are present before the motor symptoms and are about executive functions. In an individual with normal basal nuclei function, the striatum is considered the relay center for the association loops and motor loops which allow cognitive and motor behavior to be goal-directed and planned [8]. These loops also help the cerebrum determine what information is relevant and what can be ignored without an individual having to consciously think about it. In an individual with Huntington’s disease, this ability is lost and they are no longer able to organize or plan events throughout their day. There is no longer the flexibility to adjust previous plans due to their significantly decreased processing speed and inability to problem solve. This can be very frustrating for the individual and family members as they can no longer react to a situation as they might have in the past or in a way that is expected in their environment [1]. In many cases, the psychotic changes increase as the individual’s cognitive level begins to decline.

Current treatment options

Huntington’s disease is a fatal condition that currently has no cure. Once there is a demonstration of clinical symptoms, the patient will have 10 to 20 years of remaining longevity [1]. Symptomatic management options are limited at this time and do not slow the neurodegenerative process or overall rate of functional loss. Within the final pages, we discuss the symptomatic care for each of the major clinical symptoms of Huntington’s disease with a discussion of the current research methodologies and future directions.

Motor Symptoms

As mentioned above in the section titled 'Motor Changes', chorea begins as suppressible, fidgety movements in the distal extremities that include the fingers, toes, and face. As the disease progresses, these unwanted and intrusive movements involve more proximal muscles. Ballistic movements, which are rapid muscle contractions at maximal velocity, may also be visualized. This can cause a rapid weight loss due to excessive, unnecessary movement. In Grade Four of Huntington’s disease, which is the final and most advanced stage, the chorea can become so severe that there is burnout of neurons leading to bradykinesia, dystonia, and ataxia [12]. The postural instability can cause frequent falls and serious or life-threatening injuries.

Symptomatically treating chorea is only done when the excessive movements begin to impair the individual’s quality of life and safety. In many instances, the individual is not aware of the chorea and all that is needed is reassurance and education of the individual and family members. As Huntington’s disease progresses, there is initially a loss of the medium-sized densely spiny neurons of the indirect pathway. As these neurons decline, the excess glutamatergic and dopaminergic excitatory signals that would have gone to the indirect neurons are funnelled into the direct pathway. By reducing dopamine neurotransmission through presynaptic depletion or dopamine receptor blockade, there is a pharmacologic ability to reduce the excessive movement of chorea [12]. The medications that are FDA approved are tetrabenazine and deutetrabenazine (Austedo) which inhibit the vesicular monoamine transporter 2 (VMAT-2). The VMAT-2 takes serotonin, dopamine, and norepinephrine in the central nervous system and releases the neurotransmitters into the synapse when signalled. The inhibition of VMAT-2 allows for the depletion of dopamine to reduce chorea but can also lead to depleted levels of serotonin and norepinephrine. While chorea may be reduced, the depleted serotonin and norepinephrine can exacerbate symptoms of depression and anxiety. A thorough discussion between the physician and patient needs to occur to weigh the harms and benefits of pharmacological treatment.

Exercise-based therapies are non-pharmacological ways to help patients suffering from Huntington’s disease. Examples of therapies can involve physical therapists in the earlier stages to address gait impairment, occupational therapists when the chorea begins to interfere with their daily living, and social workers to help with home modifications to allow for more independence and a higher level of safety [12]. Speech pathologists can help at any stage to assist with dysarthria and dysphagia. It is highly recommended to begin speech therapy before the onset of significant dysphagia to be able to modify the diet accordingly. As mentioned previously, aspiration pneumonia is the most common cause of death in individuals with Huntington’s disease.

Psychiatric Symptoms

The psychiatric symptoms of Huntington’s disease are often the most unsettling for the individuals and their families. As mentioned above in the section titled 'Psychiatric Changes', new-onset depression, apathy, and obsessive-compulsive disorder are likely due to the dysregulation of signals from the anterior cingulate gyrus, medial and lateral temporal lobes, hippocampus, amygdala, and entorhinal area to the frontal lobe and thalamus which are normally maintained by the limbic loop of the basal nuclei [8]. These symptoms are important to recognize early as the presence of psychiatric changes has been shown to negatively correlate with the daily functioning of those affected by Huntington’s disease [12].

Depression is the most common psychiatric symptom seen in 40% to 70% of individuals with Huntington’s disease and is most commonly seen as the patient begins to lose their independence. There has been a noted benefit of using selective serotonin reuptake inhibitors (SSRIs) and the serotonin-norepinephrine reuptake inhibitor - venlafaxine - which are used as first-line agents to treat depression in non-Huntington-related related disorders [12]. These medications allow for an increased amount of serotonin and norepinephrine in the neuronal synapse due to an inhibition of the degradation process. New-onset obsessive-compulsive disorder has also responded well to SSRIs. Suicide is the second most common cause of death in those affected by Huntington's disease; therefore, aggressive treatment of these symptoms and encouragement to attend support groups are highly recommended [12].

Apathy, or lack of motivation with a decrease in goal-directed behavior, is most prevalent in the advanced stages of the disease [12]. While there are no known pharmacological treatments for apathy, it is often recommended for patients to reduce the amount of tetrabenazine if their chorea is becoming less problematic, as one of the side effects of tetrabenazine is the depletion of dopamine and serotonin, which has been seen to exacerbate psychiatric conditions as noted above.

If medications are not desired or effective, the individual may respond to a supportive, structured environment with routines and cues [12]. Not only is there concern for the individual with Huntington’s disease but there should be a frequent assessment of family members for caregiver burnout. Social workers are an essential piece to the caregiving team to help identify resources for the family whether that be care assistance, respite care, counsellors, or financial support.

Cognitive Symptoms

As discussed in the section above titled 'Cognitive Changes', the basal nuclei are considered the relay center for the association loops and motor loops to allow for planned and goal-directed cognitive behavior [8]. The cognitive effects are often misdiagnosed as this is subcortical dementia, which is considered a disease of connections. This can lead to problems with processing speed and problem solving [12]. In conditions such as Alzheimer’s disease, there is cortical dementia, which demonstrates striking and recognizable symptoms including memory loss and language problems. The cortical degeneration of Alzheimer’s disease is easily measured with screening tools such as a Mini-Mental State Exam, but these same tools would not be helpful for individuals suffering from the subcortical degeneration seen in Huntington’s disease.

The current medications used to ease cognitive impairment in Huntington’s disease are the same medications that have known benefits for Alzheimer’s dementia. The acetylcholinesterase inhibitor rivastigmine is considered the best option as it causes an increase in acetylcholine in the neuronal synapse by inhibiting its degradation process. There are few trials to test the efficacy of this medication and in these trials, there is not a definitive way to quantify the improvement or decline of patients. The common screening tools available are meant to assess the level of cortical dementia, not subcortical dementia seen in Huntington’s disease. Currently, the best treatment for an individual with Huntington’s disease is to provide supportive measures such as providing cues, minimizing the amount of multitasking, and allowing extra time to perform cognitive tasks [12].

Current research

While there are medications to ease the symptoms, they do not improve the life longevity of the patient. This is why there are many nongenetic and genetic research projects taking place to search for new treatments and possibly the cure for Huntington’s disease. The nongenetic research goals are to determine the pathophysiology behind the mutated *Huntingtin* protein and to search for reliable and detectable biomarkers. The most likely mechanism of *Huntingtin* was described in detail in the section titled 'Pathology of Mutated Huntingtin', but there is also evidence that the nonmutated function of *Huntingtin* plays a role in internal cellular signalling, prevention of apoptosis, and production of brain-derived neurotrophic factor [12]. Having medications that specifically interact with or increase the degradation of the mutated *Huntingtin* protein can drastically alleviate the suffering of individuals. The TRACK-HD study in 2009 used multi-site MRI to show quantifiable biological and clinical alternation in *Huntingtin* mutation carriers compared with age-matched controls demonstrating the feasibility of using this technique to detect Huntington's disease. [13]. They observed that there is a progressive loss seen in the gray matter and atrophy of the caudate nuclei leading to the expansion of ventricles that can be assessed by clinicians to determine the severity of the disease. 

To improve the quality of life for those with Huntington’s disease, research is being done using genetics to directly target the abnormal CAG repeats on chromosome four. By either downregulating or completely turning off the transcription and translation of mutated *Huntingtin* using clustered regularly interspaced short palindromic repeats (CRISPR) technology, scientists can stop the damaging sequelae of the mutated protein [14]. It is important to spare the normal allele of chromosome four as the wild type *Huntingtin* has essential functions of intracellular signalling and preventing apoptosis of normal cells. While this idea has been successful in animal models, it has yet to be clinically attempted in human subjects.

## Conclusions

Huntington’s disease is an autosomal dominant trinucleotide repeat disorder that demonstrates specific motor, cognitive, and psychiatric symptoms due to the atrophy of the medium-sized densely spiny neurons in the striatum. The neurons of the indirect pathway are targeted initially, which leads to hyperkinetic chorea, and eventually, the degradation of all neurons in the striatum causing akinesia, rigidity, dysarthria, and dysphagia. While Huntington’s disease was the first trinucleotide repeat disorder to be discovered, the disease is still poorly understood and there are limited treatment options. Current research on the pathogenesis of mutated* Huntingtin* and the use of CRISPR to edit the specific CAG chromosomal repeats leads to a promising future for those affected by this devastating disease.
